# A PAK6–IQGAP1 complex promotes disassembly of cell–cell adhesions

**DOI:** 10.1007/s00018-013-1528-5

**Published:** 2013-12-19

**Authors:** Sally Fram, Helen King, David B. Sacks, Claire M. Wells

**Affiliations:** 1 Division of Cancer Studies, King’s College London, New Hunts House, Guys Campus, London, SE1 1UL UK; 2 Department of Laboratory Medicine, National Institutes of Health, Bethesda, USA

**Keywords:** PAK6, IQGAP, Cell junctions, Cell motility, HGF

## Abstract

p-21 activated 6 (PAK6), first identified as interacting with the androgen receptor (AR), is over-expressed in multiple cancer tissues and has been linked to the progression of prostate cancer, however little is known about PAK6 function in the absence of AR signaling. We report here that PAK6 is specifically required for carcinoma cell–cell dissociation downstream of hepatocyte growth factor (HGF) for both DU145 prostate cancer and HT29 colon cancer cells. Moreover, PAK6 overexpression can drive cells to escape from adhesive colonies in the absence of stimulation. We have localized PAK6 to cell–cell junctions and have detected a direct interaction between the kinase domain of PAK6 and the junctional protein IQGAP1. Co-expression of IQGAP1 and PAK6 increases cell colony escape and leads to elevated PAK6 activation. Further studies have identified a PAK6/E-cadherin/IQGAP1 complex downstream of HGF. Moreover, we find that β-catenin is also localized with PAK6 in cell–cell junctions and is a novel PAK6 substrate. We propose a unique role for PAK6, independent of AR signaling, where PAK6 drives junction disassembly during HGF-driven cell–cell dissociation via an IQGAP1/E-cadherin complex that leads to the phosphorylation of β-catenin and the disruption of cell–cell adhesions.

**Electronic supplementary material:**

The online version of this article (doi:10.1007/s00018-013-1528-5) contains supplementary material, which is available to authorized users.

## Introduction

Epithelial-to-mesenchymal transition (EMT) is a developmental process that involves the loss of cell–cell junction integrity, cell–cell dissociation, and ultimately leads to the emergence of independently migratory cells [42]. Cell scattering has also been observed in some cancer cell populations where cells that have undergone an EMT-like process and are able to disseminate to distal sites [51]. Hepatocyte growth factor (HGF) is well known to elicit such a cell scattering response in a number of different cell lines, including prostate carcinoma DU145 cells [46].

In normal tissue, epithelial cells are held together by a plethora of cell–cell adhesion molecules focused around a number of different junctional complexes including tight junctions, desmosomes, and adherens junctions. During both normal and carcinoma development the initial stages of cell–cell dissociation requires the disassembly of these junctions between neighboring cells. E-cadherin is a major component of adherens junctions and loss of E-cadherin expression is often associated with cell–cell dissociation and cancer progression [8]. The homotypic interactions of E-cadherin extracellular domains are complemented by the intracellular domain of E-cadherin binding a number of cytoplasmic partners, including the catenin family of proteins, which ultimately lead to a link between E-cadherin and the actin cytoskeleton [15]. Junctional disruption is associated with a re-organization of the actin cytoskeleton, a process thought to be mediated by the activity of Rho family GTPases. While much is known about how adherens junctions are formed, less is known about the signaling events and molecular interactions that lead to junctional dissociation. Activation of Rac1 and Cdc42 can abolish the multi-cellular organization of breast carcinoma cells in a 3D matrix [20] and disrupts cell–cell adhesions in human keratinocytes [4] and pancreatic carcinoma cells [13]. Moreover, Rac1 and Cdc42 are both known to interact with isoform 1 of the IQ motif containing GTPase activating protein (IQGAP) family of proteins [21], and a Rac-1–IQGAP1 interaction is thought to mediate the dissociation of β-catenin from E-cadherin downstream of HGF in MDCK cells [10]. In addition, phosphorylation of catenin proteins has been correlated with junctional disruption [39]. However, the role of IQGAP1 in junctional dynamics is still not clearly understood.

The p-21-activated kinases (PAKs) family has been implicated in the regulation of both cell matrix adhesion and migration [49] and there is some evidence that these proteins might also play a role in junctional dynamics [29]. The family is split into two groups [49] and it has been shown that the *Drosophila* homologue of Group II PAKs is localized at adherens junctions and is involved in the cell–cell dissociation process during eye maturation [33]. A recent report also demonstrated that PAK4 interacts with β-catenin, implicating this kinase in β-catenin re-localization and signaling [26], however this study was not conducted in a colony-forming cell line so could not be correlated to junctional dynamics.

Recently, PAK6, a less well studied member of the PAK family was identified as a putative IQGAP1 binding protein [18] but the functional implications were not explored. Indeed, to date, very little is known about the role of PAK6 in mammalian cells other than as an androgen receptor-interacting protein [50]. Moreover, unlike other family members, the interaction between PAK6 and the GTP bound form of Cdc42 does not increase PAK6 kinase activity [38] and the regulatory mechanisms of PAK6 activity are not well understood. PAK6 expression has been linked to prostate cancer invasiveness but no mechanism has been identified [48]; thus a functional role for PAK6 expression outside of androgen signaling remains to be elucidated. We have used the DU145 cell scattering model to identify an essential role for PAK6 during HGF-induced cell–cell dissociation. Moreover, we find that increased levels of PAK6 expression and activity can drive cell–cell dissociation and this phenotype is further elevated upon co-expression with IQGAP1. We have characterized the interaction between IQGAP1 and PAK6 and identify IQGAP1 as a novel regulator of PAK6 kinase activity. Furthermore, we identify β-catenin as a PAK6 substrate and propose that PAK6 phosphorylation of β-catenin drives the disassembly of cell–cell adhesions.

## Results

### HGF stimulation increases PAK6 autophosphorylation

**Fig. 1:**
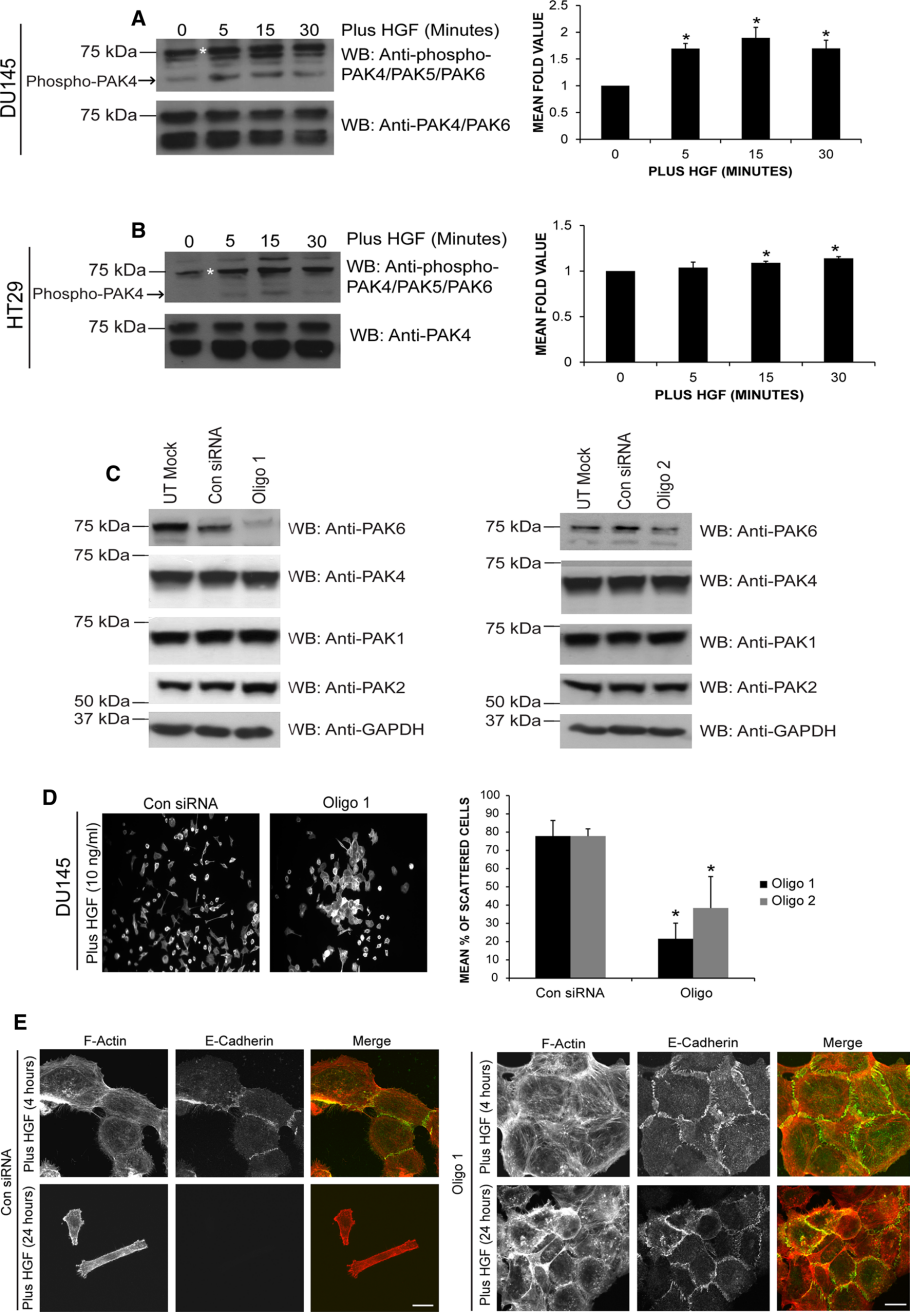
PAK6 and HGF-induced cell scattering. **a**, **b** Serum-starved cells were stimulated with HGF [10 ng/ml (**a**) or 60 ng/ml (**b**)] for the times indicated. Lysates were immunoblotted for levels of S560 PAK6 autophosphorylation. Blots were re-probed for total PAK6. Changes in PAK6 S560 levels were quantified (quantified band marked with *asterisk*) using Andor IQ software. Data represents mean ± SEM. **c** DU145 cells were transfected with control (Con) or PAK6 (Oligo 1 or 2) siRNA oligos lysed after 72 h and immunoblotted for PAK6/4/1/2 and GAPDH as a loading control. **d** DU145 cells were transfected as shown. Following 72 h, cells were serum-starved for 24 h and stimulated with HGF for 24 h. The cells were fixed and stained for F-actin, counted and scored for cell scattering. **e** DU145 cells were transfected with Con siRNA or Oligo 1. After 72 h, cells were serum-starved for 24 h and stimulated with 10 ng/ml HGF for 4 or 24 h as indicated. The cells were then fixed and stained for E-cadherin and F-actin and imaged using confocal microscopy. All data are representative of three independent experiments where *n* = 90 cells per condition. Data represents mean ± SEM. Statistical significance was calculated using Student’s *t* test, *p < 0.05

We have previously used a HGF-induced human cell scattering model to elucidate the role of PAK4 in adhesion turnover [47]. We now report that colon carcinoma HT29 cells (express c-Met) can also be used to monitor HGF-induced cell scattering (Figs. S1A and S1B). Our previous work had utilized an antibody that recognizes both PAK4 and PAK6 to detect PAK6 in DU145 cells [47] (hereafter referred to as anti-PAK4/PAK6) (Fig. S1C) we now incorporate the use of a PAK6-specific antibody (Calbiochem). Using these antibodies, we can confirm that PAK6 is expressed in DU145 and HT29 cell lines (Fig. S1D). Serine 560 (S560) in the PAK6 kinase domain is thought to be an autophosphorylation site homologous to serine 474 (S474) in PAK4 [1]. Upon HGF stimulation in both DU145 and HT29 cells, endogenous PAK6 S560 levels were seen to significantly increase (Fig. 1a, b). An increased concentration of HGF was required to elicit the HT29 response (which was less than that seen in DU145 cells), which may be due to accessibility of c-Met receptor within the tightly packed HT29 cell colonies.

### PAK6 is required for HGF-induced cell–cell dissociation

Given that PAK6 autophosphorylation is elevated downstream of HGF it might be speculated that PAK6 plays a role in the cellular response to HGF. Two different siRNA oligonucleotides were employed to knockdown PAK6 expression levels in DU145 cells (Figs. 1c, S1E). The level of PAK6 was significantly reduced when compared to control siRNA lysates and neither siRNA affected PAK1, PAK2, or PAK4 expression (Fig. 1c). DU145 cells transfected with control siRNA exhibited HGF-induced cell–cell dissociation and subsequent cell scattering, by contrast loss of PAK6 expression significantly reduced the scattering response of both DU145 and HT29 cells to HGF (Figs. 1d, S2A). Closer examination of HGF-treated PAK6 siRNA colonies suggested that cells were not disassembling their cell–cell junctions, a normal HGF response (Fig. 1e). In control siRNA-treated cells following 4 h of HGF stimulation cell–cell adhesions can be seen to dissociate and E-cadherin staining (a junctional marker [12]) is only present at adjoining cells. At 24 h after HGF stimulation, E-cadherin-positive staining is undetectable in the control cells. In contrast, the cell borders in PAK6 knockdown cell populations were E-cadherin positive, with distinct and extensive punctate staining following 4-h stimulation with HGF (Fig. 1e) and the cell–cell boundaries in the PAK6 knockdown cell population were still robustly E-cadherin positive after 24 h of stimulation (Fig. 1e).

### PAK6 kinase activity drives cell colony escape/extrusion

**Fig. 2:**
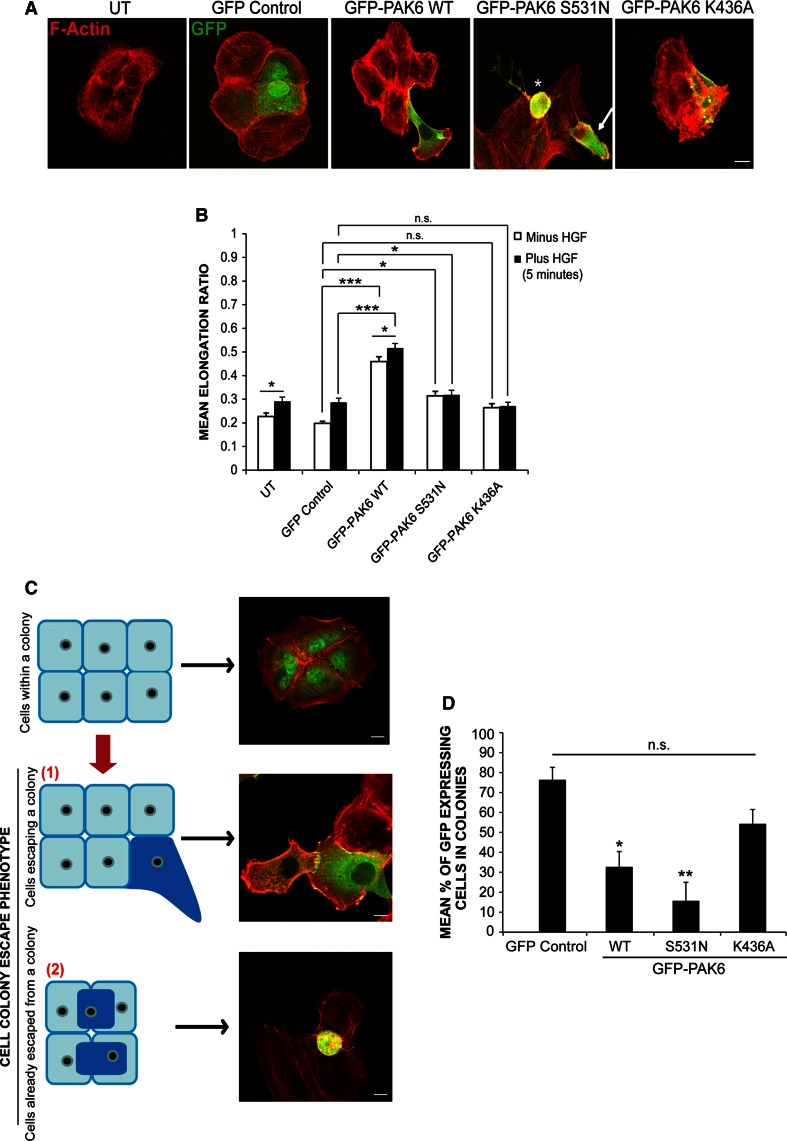
PAK6 overexpression induces morphological changes in DU145 cells. **a,** **b** DU145 cells were transfected with GFP control vector or GFP-PAK6 mutants as indicated. Serum-starved cells were stimulated with HGF (plus HGF) for 5 min. Cells were then fixed and stained for F-actin and imaged using confocal microscopy. Shape analysis was performed on the cells using ImageJ software to determine the elongation ratio. *Asterisk* PAK6^S531N^-expressing cell with rounded phenotype. *Arrow* PAK6^S531N^-expressing cell with elongated phenotype. **c** Schematic illustrating cell phenotype categorization *1* cells escaping a colony defined as greater than 50 % of the cell body perimeter detached from the neighboring cell(s). *2* cells already escaped from a colony defined as cells exhibiting 100 % dissociation from the neighboring cell(s) or cells in a different plane to the underlying cell colony. **d** The mean % of GFP-expressing cells present in colonies. Data represents 110 cells per condition over three independent experiments, mean ± SEM. Statistical significance compared with GFP control cells was calculated using Student’s *t* test, *p < 0.05, **p < 0.005. ***p < 0.0005, *ns* not significant. *bar* = 10 μm

Given that cells with reduced PAK6 expression are unable to dissemble junctions in response to HGF, we wondered whether PAK6 might promote junction disassembly. To investigate the effect of PAK6 overexpression, a number of PAK6 derivatives were generated; full-length PAK6 (PAK6^wt^), full-length kinase dead PAK6 (PAK6^K436A^) and full-length kinase activated PAK6 (PAK6^S531N^). All derivatives were tested for binding to Cdc42 and for the level of kinase activity (Figs. S2B and C). It was immediately apparent that PAK6 overexpression induced a distinctive phenotype when compared to control cells (Fig. 2a, b) where PAK6^wt^-expressing cells were significantly elongated and highly expressing cells become rounded. PAK6 ^S531N^-expressing cells were also elongated, although not to the same degree as WT PAK6-expressing cells (Fig. 2a, b), and highly expressing cells also become rounded. However, cells expressing PAK6^K436A^ were not significantly different to control cells (Fig. 2a, b). Upon closer observation, it was clear that cells overexpressing PAK6 were detaching from neighboring cells and were no longer contained within the cell colony. Moreover, PAK6^S531N^ cells were being extruded from cell colonies (Fig. 2a, arrow) while there was no obvious dissociation of PAK6^K346A^ cells (Fig. S2E). To further investigate this colony escape/extrusion phenomenon, expressing cells were scored based on whether they were present in a cell colony versus escaping the cell colony (Fig. 2c). While control GFP-expressing cells clearly remained in colonies, the percentage of PAK6^wt^ and PAK6^S531N^-expressing cells retained in colonies was significantly reduced (Fig. 2d). In contrast, cells expressing PAK6^K436A^ were found predominantly within a cell colony (Fig. 2d). Over-expression of PAK6^wt^ in HT29 cells also drove colony escape to a significant level when compared to GFP control cells (Fig. S2D).

### PAK6 localizes to cell–cell junctions

**Fig. 3:**
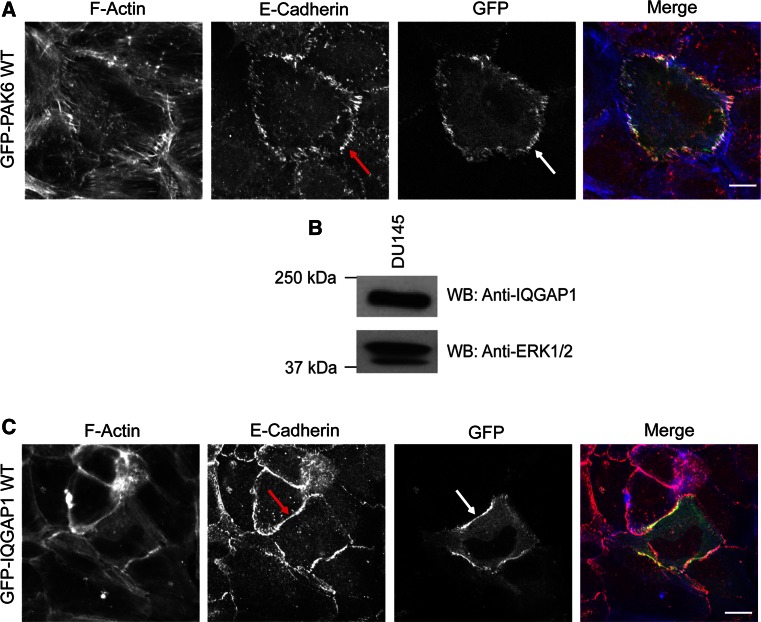
PAK6 and IQGAP1 at cell–cell junctions. **a**, **c** DU145 cells were transfected with GFP-PAK6^wt^ or IQGAP1-GFP^wt^. Serum-starved cells were fixed and labeled for E-cadherin and F-actin. Low-level GFP-PAK6^wt^ and IQGAP1-GFP^wt^ expression could be detected at E-cadherin-positive cell–cell junctions (*arrows*). **b** DU145 whole-cell lysate was immunoblotted for endogenous IQGAP1 and for ERK1/2 as a loading control. All data are representative of three independent experiments. *bar* = 10 μm

Our data suggest that PAK6 can regulate the integrity of cell–cell adhesions. Due to the lack of appropriate antibody, we cannot detect endogenous PAK6, thus cells expressing a low level of PAK6^wt^ were targeted (cells with high PAK6^wt^ expression are escaping the cell colony). PAK6 was found to be specifically localized at cell–cell boundaries with punctate and distinct localization (Fig. 3a), which correlated with the pattern of E-cadherin localization (Fig. 3a, arrows). The localization of PAK6^S531N^ could not be determined in colony cells as PAK6^S531N^-expressing cells had already escaped from colonies, but PAK6^K436A^ was diffusely localized in the cell cytoplasm and was never specifically detected at cell junctions (Fig. S2E).

### PAK6 interacts with IQGAP1

To further explain the mechanism underlying PAK6 mediation of junctional integrity, we sought to identify junction-relevant PAK6 binding partners. A previous analysis by mass spectrometry of protein obtained from 293-T cells identified IQGAP1 as a possible PAK6-interacting protein [18]. IQGAP1 is thought to regulate cell–cell dissociation downstream of HGF [10]. Thus, we speculated that PAK6 and IQGAP1 might play a synergistic role in disruption of cell–cell adhesions. Although IQGAP1 is thought to be ubiquitously expressed [44], its expression in prostate cancer cells has not been examined. We found that IQGAP1 was expressed in DU145 cells (Fig. 3b). Moreover, overexpressed IQGAP1^wt^ was detected and enriched at E-cadherin-positive junctions (Fig. 3c), consistent with previous studies in breast epithelial cells [28, 41].

**Fig. 4:**
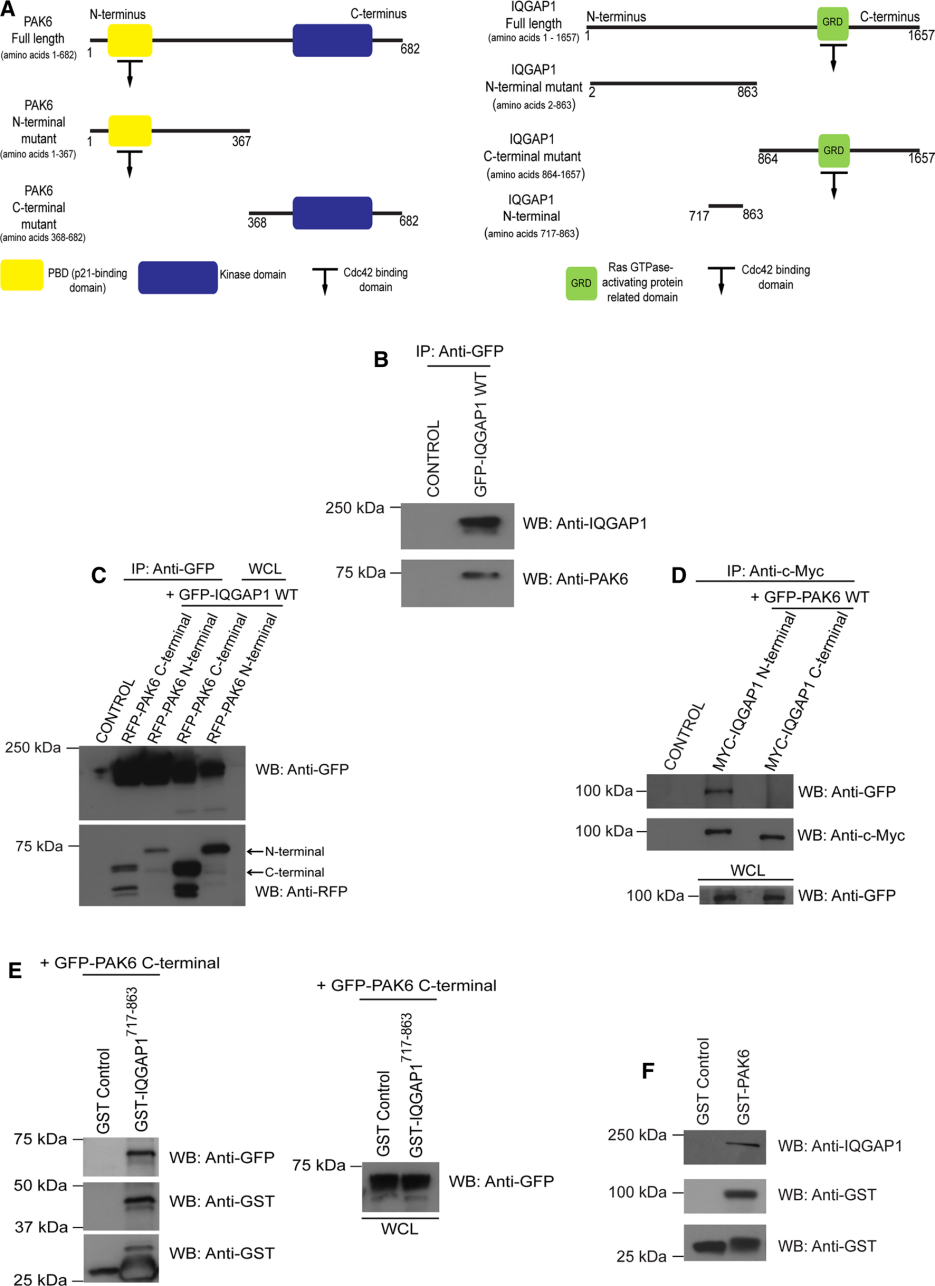
**a** PAK6 and IQGAP1 domain interactions. **a** Schematic illustrating PAK6 and IQGAP1-generated constructs. **b** HEK293 cells were transfected with IQGAP1-GFP^wt^. Samples were immunoprecipitated with anti-GFP antibodies and immunoblotted for endogenous PAK6 and IQGAP1-GFP^wt^. **c** HEK293 cells were transfected with N- or C-terminal RFP-PAK6 mutants and IQGAP1-GFP^wt^ as indicated. Immunoprecipitated IQGAP1-GFP^wt^ samples were immunoblotted using anti-GFP and anti-RFP antibodies. Whole-cell lysates (WCL) were immunoblotted using anti-GFP and anti-RFP antibodies as controls (*arrows*). **d** HEK293 cells were transfected with N- or C- terminal Myc-IQGAP1 mutants and GFP-PAK6^wt^ as indicated. Immunoprecipitated N- and C-terminal IQGAP1 samples were immunoblotted for GFP-PAK6^wt^ and for N- and C-terminal Myc-IQGAP1. WCL were immunoblotted using an anti-GFP antibody as a loading control. **e** HEK293 cells were transfected with C-terminal GFP-PAK6 and lysates used in a GST or GST-IQGAP1^717–863^ pulldown. Samples were immunoblotted with anti-GFP and anti-GST antibodies. WCL were immunoblotted with anti-GFP antibody as a control. **f** GST-PAK6 and GST alone pulldowns from DU145 cell lysates were immunoblotted for endogenous IQGAP1 and GST. Data are representative of three independent experiments, control = untransfected cells

To further characterize the putative interaction between IQGAP1 and PAK6, a structure–function analysis was conducted in HEK293 cells using various full-length and domain mutants (Fig. 4a). Here we were able to show that endogenous PAK6 and GFP-IQGAP1^WT^ are immunoprecipitated together (Fig. 4b). Both PAK6 and IQGAP1 are reported to interact with activated Cdc42 [14, 21, 24]. Thus, the interaction between IQGAP1 and PAK6 may be mediated through a joint interaction with Cdc42. Indeed, full-length IQGAP1^wt^, Cdc42^v12^, and full-length PAK6^wt^ were present in a complex (Fig. S3A). However, we also detected IQGAP1^wt^ bound to the C-terminus of PAK6 where there is no Cdc42 binding site (Fig. 4c), and full-length PAK6^wt^ selectively interacted with the N-terminal region of IQGAP1 (Fig. 4d) outside the Cdc42 binding region, suggesting the interaction can be Cdc42 independent. Indeed using IQGAP1 N-terminal truncated proteins we were able to specify that the C-terminus of PAK6 binds to IQGAP1^717–863^ (Fig. 4e). IQGAP1^717–863^ does not contain the Cdc42 binding site [14, 31], confirming that Cdc42 is not required. Subsequently, we were able to detect the interaction in DU145 cells where GST-PAK6 bound to endogenous IQGAP1 (Fig. 4f).

### IQGAP1 induces colony escape synergistically with PAK6

**Fig. 5:**
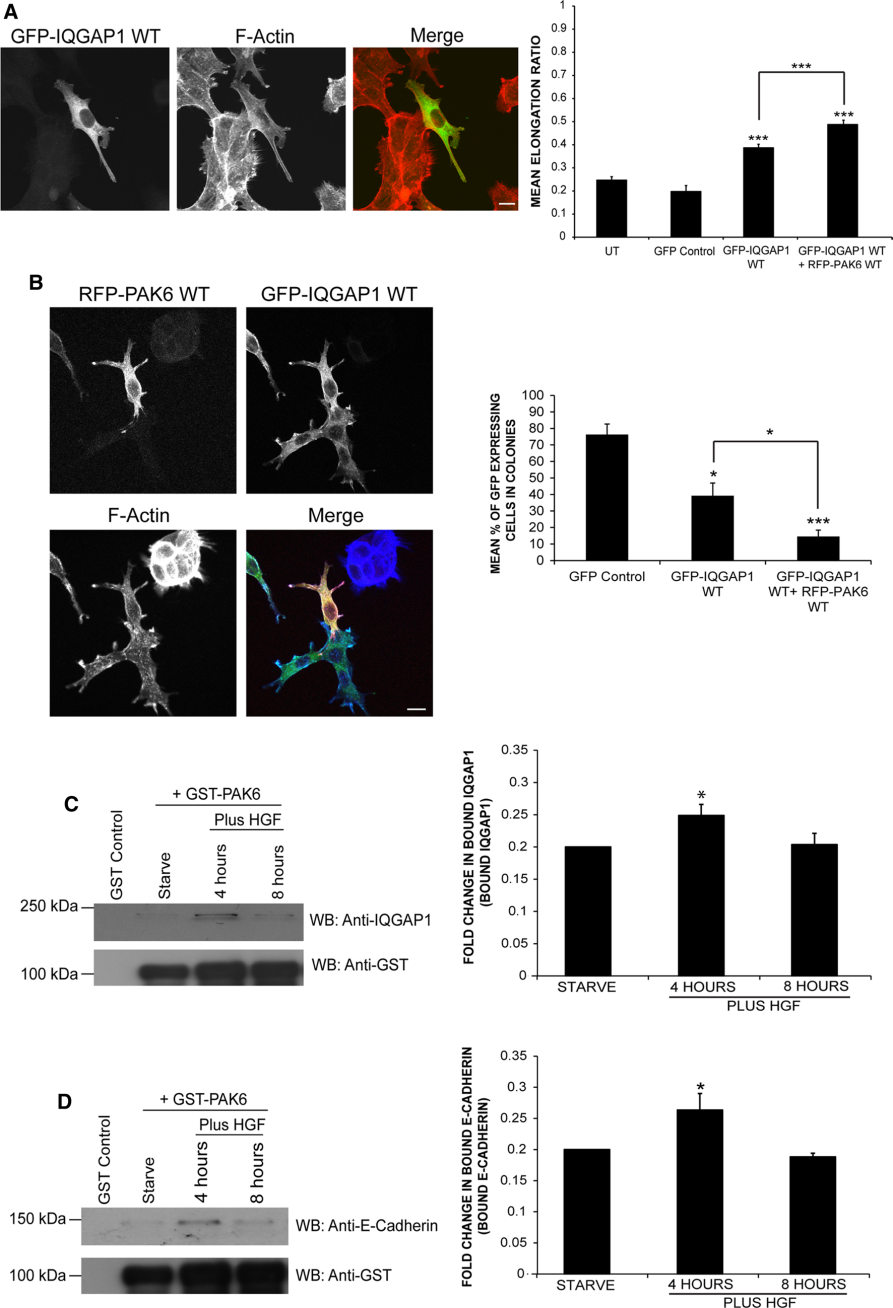
IQGAP1 overexpression induces morphological changes in DU145 cells. **a**, **b** DU145 cells were transfected as indicated or left as untransfected cells (UT). Serum-starved cells were then fixed and stained for F-actin. Shape analysis was performed on the cells using ImageJ software to determine the elongation ratio. Cells were then scored for presence within a colony compared to control cells (as described in Fig. 2). 110 cells were analyzed per condition over three independent experiments. **c**, **d** GST-PAK6 or GST was used in a pulldown from serum-starved or HGF (250 ng/ml) stimulated DU145 cell lysates as indicated. Pull-down samples were immunoblotted for endogenous IQGAP1 (**c**) or endogenous E-cadherin (**d**). The level of binding between PAK6 and IQGAP1 was quantified using ANDOR IQ Technology software. Data represents mean ± SEM. Statistical significance was calculated using Student’s *t* test; *p < 0.05, ***p < 0.0005. *bar* = 10 μm

Given that IQGAP1 and PAK6 interact, the effect of IQGAP1^wt^ overexpression on cells was compared to the morphological changes already described for PAK6 (Fig. 2a, b). GFP-IQGAP1^wt^-expressing cells exhibited significantly higher elongation ratios than control cells (Fig. 5a), similar to the cell shape changes observed for PAK6-expressing cells. Moreover, when GFP-IQGAP1^wt^ was co-expressed with RFP-PAK6^wt^ the phenotype was further enhanced (Fig. 5a, b). Moreover, IQGAP1^wt^-expressing cells and cells co-expressing IQGAP1^wt^ and PAK6^wt^ were uncoupled from neighboring cells and were no longer within the cell colony (Fig. 5b) thereby replicating and enhancing the PAK6^wt^ phenotype, respectively.

### Peak interaction between PAK6 and IQGAP1 following 4-h HGF stimulation

Loss of E-cadherin positive cell–cell junctions from DU145 cells responding to HGF occurs approximately 4 h post-stimulation (Fig. 1e). A small but significant increase in the interaction between GST-PAK6 and endogenous IQGAP1 was observed following 4-h HGF stimulation when compared to serum-starved conditions (Fig. 5c), thereby correlating with HGF-induced cell–cell dissociation. Furthermore, following 8-h stimulation, the interaction between these two proteins was restored to near serum-starved levels (Fig. 5c). In PAK6 knockdown cells, E-cadherin is retained at cell–cell junctions downstream of HGF (Fig. 1e). We found that GST-PAK6 could specifically pulldown E-cadherin, moreover following 4-h HGF stimulation, there was a significant increase in the level of interaction between GST-PAK6 and endogenous E-cadherin (Fig. 5d). Additionally, the interaction between GST-PAK6 and E-cadherin diminished following 8-h HGF stimulation, similar to the interaction trend observed between GST-PAK6 and IQGAP1 (Fig. 5c). Furthermore, GST- PAK6, endogenous IQGAP1, and endogenous E-cadherin were found in a complex together following 4-h HGF stimulation (Fig. S3B).

### PAK6 phosphorylation levels are elevated in the presence of IQGAP1

**Fig. 6:**
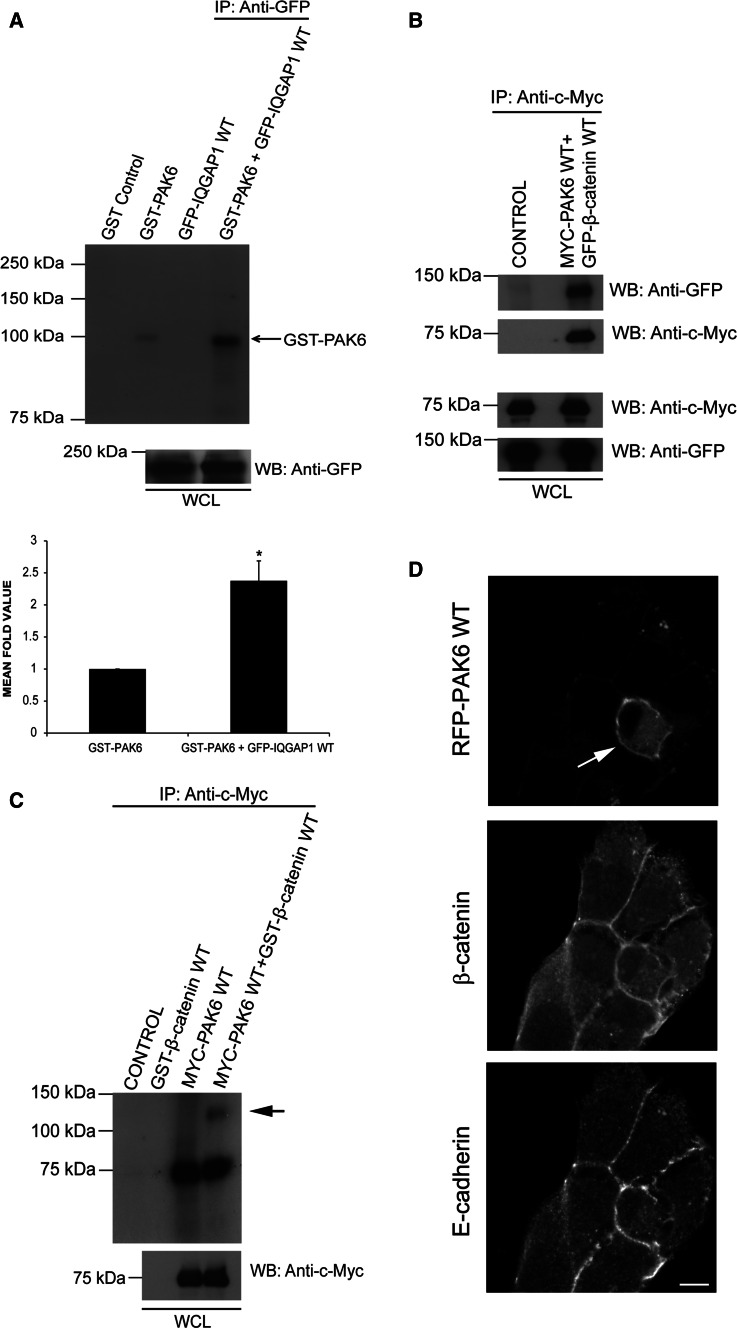
IQGAP1 expression increases PAK6 phosphorylation levels. **a** GFP-IQGAP1^wt^ was immunoprecipitated from HEK293 cells and mixed with or without GST-PAK6 as indicated. An in vitro kinase assay was performed using [γ-^32^P] ATP. GST-PAK6 alone was also subjected to the in vitro kinase assay and GST was used as a control. WCL were immunoblotted using an anti-GFP antibody as a loading control. Quantification indicates mean fold increase in autophosphorylation signal between GST-PAK6 alone and GST-PAK6 in the presence of GFP-IQGAP1^wt^. **b** HEK293 cells were transfected as indicated or left as untransfected cells (control) samples were immunoprecipitated with anti-c-Myc antibodies and immunoblotted for GFP-β-catenin^wt^ and Myc-PAK6^wt^ as a loading control. WCL were immunoblotted for GFP and Myc as a control. **c** Myc-PAK6^wt^ was immunoprecipitated from cell lysates and mixed with or without WT GST- β-catenin as indicated. An in vitro kinase assay was performed using [γ-^32^P] ATP. GST- β-catenin alone was also subjected to the in vitro kinase assay. WCL were immunoblotted using an anti-c-Myc antibody as a loading control. **d** WT RFP-PAK6-transfected DU145 cells were stained for endogenous β-catenin and for endogenous E-cadherin. *bar* = 10 μm. All data are representative of three independent experiments, mean ± SEM

Our data points to an E-cadherin, IQGAP1, and PAK6 complex that forms as cell–cell dissociation occurs. As IQGAP1 binds to the kinase region of PAK6 (Fig. 4e), it was hypothesized that IQGAP1 may be a PAK6 substrate. However, in an in vitro kinase assay, recombinant PAK6 did not phosphorylate IQGAP1^wt^ (Fig. 6a). Unexpectedly, the presence of IQGAP1^wt^ induced an increase in the autophosphorylation level of PAK6 (Fig. 6a). There are no other detectable bands in the in vitro kinase assay so we do not think that another kinase/activating proteins are being co-immunoprecipitated with IQGAP1. Although PAK6 S560 levels were modestly increased upon early HGF stimulation (Fig. 1a, b), the phosphorylation levels at this residue were unaltered in the presence of the IQGAP1 (Fig. S3C), implying that IQGAP1 affects other sites. Moreover, S560 levels were not elevated during the peak interaction of IQGAP1, E-cadherin and PAK6 downstream of HGF (Fig. S3D), and an S560 point mutation does not elevate the kinase activity of PAK6 (Fig. S2C). Thus, the full significance of S560 phosphorylation remains to be elucidated.

### PAK6 binds to and phosphorylates β-catenin

**Fig. 7:**
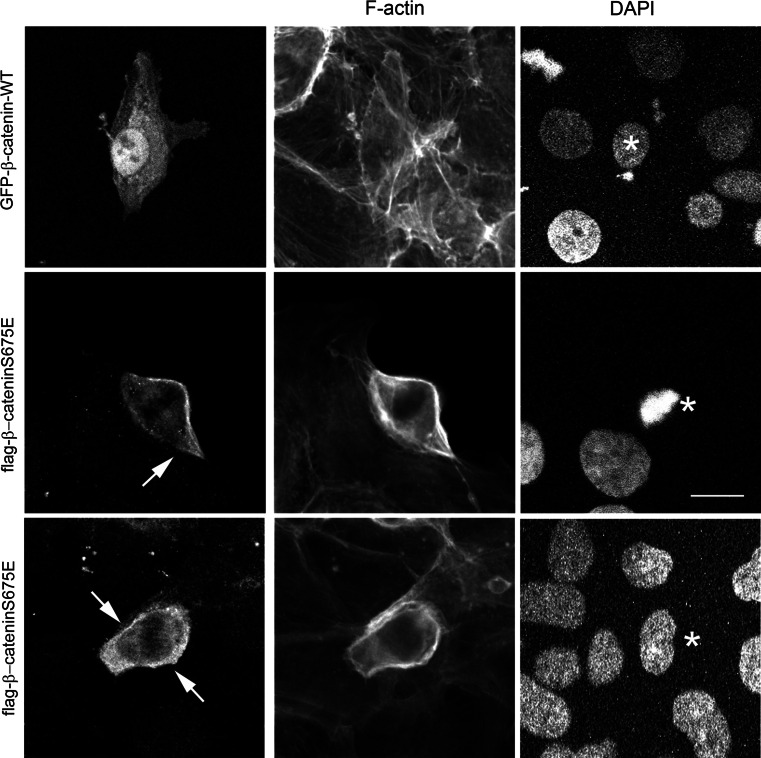
β-catenin S675A preferentially localizes to the cell periphery. DU145 cells were transfected with GFP-β-catenin^wt^ or flag-β-catenin^S675E^ as indicated. Cells were fixed and stained for F-actin and flag tag as required. All data are representative of three independent experiments. *Arrow* indicates the peripheral localization of β-catenin^S675E^, and the *asterisk* indicates position of expressing cell nucleus within the colony. *Bar* = 10 μm

Given that IQGAP1 is not a PAK6 substrate, we looked for alternative substrates in the context of cell–cell junctions. IQGAP1 and E-cadherin both interact with β-catenin [5, 22]. Moreover, PAK1 and PAK4 have been shown to phosphorylate β-catenin at serine 675 (S675), although this was associated with Wnt signaling and transcriptional activation [26, 40]. Interestingly, the *Drosophila* homologue of PAK4/PAK6 destabilizes junctions via phosphorylation of armadillo, a β-catenin homologue [33]. We find here that PAK6 can be immunoprecipitated with β-catenin from cell lysates (Fig. 6b). Furthermore, in an in vitro kinase assay, PAK6 can directly phosphorylate β-catenin (Fig. 6c, arrow). Importantly, PAK6 and β-catenin are both localized at E-cadherin-positive cell junctions (Fig. 6d). Moreover, in an in vitro kinase assay using recombinant proteins, the level of β-catenin serine 675 phosphorylation is increased in the presence of PAK6 (Fig. S3E). These data demonstrate that PAK6 phosphorylates β-catenin, and allowed us to hypothesize that serine 675 phosphorylation might be linked to junctional dissociation. To further test this hypothesis, we have overexpressed β-catenin^wt^ and non-phosphorylatable β-catenin^S675A^ in our cells. Interestingly, we find that that while overexpressed β-catenin^wt^ is predominantly nuclear with some cytoplasmic localization, overexpression of β-catenin^S675A^ is consistently found at the cell periphery in areas of cell–cell association (Fig. 7 arrow), and is often excluded from the nucleus, suggesting that phosphorylation at S675 facilitates the removal of β-catenin from junctional complexes. The differential localization of β-catenin^wt^ compared to β-catenin^S675A^ was further validated by performing the analysis in a second colony-forming cell line (Fig. S4).

## Discussion

Very little is known about the function of PAK6 in cells other than as an androgen receptor binding protein or in the context of neuronal cell development. We report here that depletion of PAK6 expression inhibits HGF-induced cell–cell junction dissolution and subsequent cell scattering. In contrast, overexpression of PAK6 drives cell–cell dissemination, a process dependent on kinase activity. PAK6 was localized at E-cadherin-positive junctions and was found to interact with the junctional protein IQGAP1, with increased interaction downstream of HGF. Thus, we propose that PAK6 and IQGAP1 function synergistically downstream of HGF to induce cell–cell dissociation.

Cell–cell dissociation is both a normal physiological process during embryonic development and part of the pathological progression of cancer. However, while the molecular mechanisms that drive cell–cell adhesion formation are well characterized, the dissolution of junctions is less well understood. It is well established that HGF can induce cell–cell dissociation and that HGF signaling plays a role in prostate cancer metastasis [11]. We have found that HGF stimulation only modestly increases the level of S560 PAK6 autophosphorylation and S560 phosphorylation was still detected in serum-starved conditions. Recent evidence does suggest that the comparable serine site in PAK4 (S474) might be constitutively phosphorylated in some cells [3]. However, PAK4 S474 levels are suppressed by serum starvation and re-elevated by exposure to HGF in DU145 cells (this paper and previous work [45, 47]. PAK1, the most extensively studied group I PAK, is known to have multiple autophosphorylation sites [7]. It is possible that PAK6 is phosphorylated downstream of HGF on residues other than serine 560, which has been tested here, and thus these results may not reflect the complete activating potential of HGF signaling on PAK6. Indeed, we observed that mutating serine 560 does not increase the kinase activity of PAK6, but that mutating serine 531 increased PAK6 kinase activity (reported in previous work [38]). In addition, while serine 560 phosphorylation is known to be required for PAK6 activation via MKK6, PAK6 is also directly activated by MKK6 at tyrosine 566 [19]. This supports a hypothesis that the phosphorylation of other residues on PAK6 is important for PAK6 activation.

During HGF-induced DU145 cell scattering, cells become more elongated, undergo cell–cell detachment, and subsequent cell colony escape [46]. PAK6 overexpression was found to induce such morphological and phenotypical changes in DU145 cells in the absence of HGF stimulation. This is in contrast to overexpression of other PAK family members, where PAK4 overexpression exhibited no effects on the morphology of MDCK cells [45] or NIH 3T3 mouse embryo fibroblasts [36]. Indeed, the majority of PAK-induced cell-shape changes have been detected only when the constitutively active forms of PAK1, 2, or 4 were utilized [30, 36, 53]. Not only did overexpression of PAK6^wt^ induce cell elongation, but it also drove escape from a cell colony in the absence of HGF stimulation. Moreover, colony escape was dependent on PAK6 kinase activity. Colony-escape mechanisms have been reported in epithelial cell colonies during epithelial cancer cell outgrowth where aberrant proliferation of these cells facilitates cancer cell progression [25]. Moreover, MDCK cells expressing oncogenic Ras basally extrude from epithelial cell sheets [17].

The data presented here strongly suggest that PAK6 plays a role in cell–cell dissociation in different colony-forming cancer cell types. Among the PAK family members tested, this phenotype is unique to PAK6 as depletion of PAK4 did not inhibit junction disassembly [47] and depletion of PAK1/PAK2 did not result in the retention of predominant E-cadherin-positive junctions [6]. Interestingly, Mbt, a *Drosophila* PAK protein that shares close homology with human group II PAKs, localizes at adherens junctions when activated, and has been reported to induce the breakdown of these junctions during eye maturation [33]. In addition, the *Xenopus* PAK4 homologue, X-PAK5, also localizes at sites of cell contact and has been implicated in the cell–cell dissociation process [9]. We would speculate that in both of these instances the functionality could be related to PAK6 rather than other group II proteins.

Consistent with a role in junctional dynamics, we find PAK6 localized to cell–cell adhesions, and co-localized with E-cadherin and IQGAP1 at these regions. Stimulation of cells with HGF leads to an increased association between IQGAP1 and PAK6, revealing that HGF modulates the interaction. In our studies, we found that the C-terminal kinase domain of PAK6 independently interacted with the N-terminal of IQGAP1, a region that binds to the kinase domain of the EGFR [32]. IQGAP1 modulates the activation of the EGFR and subsequent signaling through this association [32]. We found that the presence of IQGAP1 increased the levels of PAK6 autophosphorylation but did not lead to phosphorylation of IQGAP1. This is consistent with a recent report that overexpression of IQGAP1 increases Aurora-A phosphorylation levels [52]. However, the increased PAK6 autophosphorylation detected is unlikely to be focused on S560, as we found no evidence that co-expression of IQGAP1 and PAK6 modulates phosphorylation levels at this specific site. Moreover, S560 levels downstream of HGF did not increase to a significant level after 4-h HGF addition, when IQGAP1 and E-cadherin interactions with PAK6 are maximal.

PAK6, IQGAP1, and E-cadherin were found in complex downstream of HGF at a time point concomitant with cell–cell dissociation. IQGAP1 also localizes at cell–cell junctions in MCF-7 breast cancer cells [41] and the increased junctional localization of this protein correlates with a reduction in E-cadherin localization at sites of cell–cell contact in breast cancer cells [28]. Indeed, IQGAP1 has been shown to negatively modulate cell–cell adhesion in MDCK II colony-forming cells downstream of HGF [10]. However, the mechanism whereby IQGAP1 drives junction disassembly is not clearly elucidated. Historically, IQGAP1 was thought to contribute to a reduction in cell–cell adhesivity through its association with β-catenin [22]. It was proposed that this interaction with β-catenin induces α-catenin displacement from the E-cadherin-β-catenin complex in vitro and in vivo [22] and that removal of α-catenin weakens these adhesions [22, 35], thereby inducing cell scattering [10]. Interestingly, studies have shown that α-catenin expression is lost from gastric, breast, and lung adenocarcinoma cells, cell types that also exhibit a scattered phenotype [34, 43].

Our data identify β-catenin as a novel substrate for human PAK6. The hyper-phosphorylation of β-catenin on serine/threonine residues has been shown to induce the loss of cell–cell junction sites from human epidermal cells [39], while a reduction in the phosphorylation of β-catenin promotes cell–cell adhesion [23]. PAK1 [54] and PAK4 [27] are both reported to phosphorylate β-catenin at serine 675 and we now find that the presence of PAK6 can also lead to increased levels of S675 phosphorylation. Moreover, our over-expression studies suggest that the phosphorylation status of β-catenin S675 correlates with a change in subcellular localization where β-catenin^wt^ is more likely to be found in the cell nucleus, consistent with previous findings [40], while a β-catenin mutant that cannot be phosphorylated at S675 (β-catenin^S657A)^ is more likely to be held in the cell–cell junction region of the cell.

We propose that after HGF stimulation there is an optimal interaction between E-cadherin, PAK6, and IQGAP1, which plays a role in driving cell–cell dissociation. Interaction between PAK6 and IQGAP1 elevates PAK6 activity, which allows for β-catenin serine phosphorylation. These events could then trigger α-catenin dissociation from the E-cadherin-β-catenin complex, thereby inducing cell–cell dissociation.

## Materials and methods

### Antibodies and reagents

Anti-PAK4 (cross-reacts with PAK6) [47], anti-phospho-PAK4/5/6 (S560), anti-PAK1, anti-PAK2, anti-IQGAP1, anti-ERK1/2, and anti-Cdc42 were obtained from Cell Signaling Technology. Anti-GAPDH was obtained from Millipore, anti-PAK6 from Calbiochem, anti-GFP from Roche, anti-RFP from Living Colors, Clontech, anti-E-cadherin (HECD-1) from GeneTex. Anti-c-Myc (9E10), c-Met (C-12), anti-HA, and anti-Flag were obtained from Sigma-Aldrich. Recombinant human HGF was purchased from R&D Systems. WT GST-β-catenin was obtained from Abcam. GFP-IQGAP1^wt^, GFP-IQGAP1 dominant negative, Myc-IQGAP1 N- and C-terminal mutants and GST-IQGAP1^717–863^ are previously described [16, 37]. HA-Cdc42^v12^ was a kind gift from Maddy Parsons, King’s College London. GFP-β-catenin^wt^ was a kind gift from Mark Dodding, King’s College London. Flag-β-catenin^S675E^ and Flag-β-catenin^S675A^ and Myc-PAK6^wt^, were a kind gift from Jonathan Chernoff, Fox Chase Cancer Center, Philadelphia, PA, USA. The Gateway Technology system (Invitrogen) was used to generate tagged PAK6 constructs Myc-PAK6^wt^ was used as the DNA template in the production of PAK6 DNA flanked by *att*B sequences; the addition of *att*B sequences is required for subsequent cloning into Gateway vectors. PAK6 DNA flanked by *att*B sequences was generated by PCR amplification using PAK6-specific primers: *att*B1 forward primer: 5′**-**GGGG ACA AGT TTG TAC AAA AAA GCA GGC TTG ATG TTC CGC AAG AAA AAG AAG AAA**-**3′. *att*B2 reverse primer: 5′**-**GGGG A CCA CTT TGT ACA AGA AAG CTG GGT C TCA GCA GGT GGA GGT CTG CTT TCG-3′. The PCR products were used in Gateway recombination to generate entry clones that were sequenced prior to further recombination to generate expression vectors encoding GST-PAK6 [using pDEST15 (Invitrogen)], GFP-PAK6^wt^, GFP-PAK6 C-terminal mutant, RFP-PAK6 N- and C-terminal mutants and RFP-PAK6^wt^. The fidelity of these plasmids was subsequently confirmed by sequencing. Plasmid pENTR-PAK6 was used as a template for the mutagenic reactions. Clones were screened by sequencing and aligned to wild-type sequences to confirm mutagenesis, prior to Gateway (Invitrogen) recombination to generate a expression vectors encoding GFP-PAK6^S531N^, GFP-PAK6^S560E^, and GFP-PAK6^K436A^.

### Cell culture

DU145 cells (ETCC) and Capan-1 cells (ETCC) were grown in RPMI-1640 (Sigma), supplemented with 10 % FBS (Helena Biosciences), l-glutamine, and 100 U/ml penicillin–streptomycin. In all cases, pre-plated cells were serum-starved for 24 h in low-serum media consisting of RPMI-1640 supplemented with 0.5 % FBS, l-glutamine, and 100 U/ml penicillin–streptomycin prior to HGF stimulation. DU145 cells were transiently transfected using Fugene-6 HD transfection reagent according to the manufacturer’s protocol (Roche). HEK293 (ETCC) were grown in DMEM-GlutaMAX (Sigma) supplemented with 10 % FBS, l-glutamine, and 100 U/ml penicillin–streptomycin, and transfected by calcium-phosphate transfection according to the manufacturer’s protocol (Invitrogen). HT29 cells (ETCC) were grown in DMEM (Sigma) supplemented with 10 % FBS, l-glutamine, and 100 U/ml penicillin–streptomycin. HT29 cells were transiently transfected using Lipofectamine 2000 according to the manufacturer’s protocol (Invitrogen).

### Knockdown of PAK6 expression

*PAK6* siRNA oligonucleotide 1 (Oligo 1) was purchased from Ambion (Austin, TX, USA). The sense sequence was 5′-GGCUAUUCCGAAGCAUGUUtt-3′. *PAK6* siRNA oligonucleotide (Oligo 2) was purchased from Thermo Scientific Dharmacon, UK. The sense sequence was 5′-CCAAUGGGCUGGCUGCAAA-3′. Control-RNA oligonucleotide was purchased from Qiagen (cat. no. 1022076). Control and *PAK6*-specific oligos were added to cells using HiPerFect Transfection Reagent (Qiagen) according to the manufacturer’s instructions to a final concentration of 75 nM (Oligo 1) and 225 nM (Oligo 2).

### Immunofluorescence and image analysis

Cells were fixed with 4 % PFA:PBS at room temperature for 20 min, washed three times with PBS, and permeabilized with 0.2 % Triton X-100/PBS for 5 min. Cells were then washed three times with PBS. For F-actin alone, cells were incubated at room temperature for 1 h with Phalloidin (Sigma). Coverslips were washed with PBS and mounted. For E-cadherin staining, cells were blocked in 3 % BSA-PBS for 30 min, washed three times with PBS, and incubated at room temperature for 2 h with anti-E-cadherin. Cells were then washed three times with PBS and incubated with anti-mouse secondary antibodies in addition to phalloidin. Coverslips were then washed and mounted. Images were collected on an Olympus IX71 inverted microscope, or a Carl Zeiss LSM510 META laser scanning confocal microscope. Images were processed in Adobe Photoshop CS5. Cell area and elongation ratio [2] were quantified using ImageJ software (NIH).

### Immunoprecipitation

Cells were lysed as previously described [45]. For immunoprecipitation experiments, cell lysates were pre-cleared with IgG coupled Protein A or G Sepharose beads (GE Healthcare) for 1 h at 4 °C. The pre-cleared lysates were then mixed with primary antibody overnight at 4 °C followed by 1-h incubation with protein A or G-Sepharose beads. The immune complexes were washed three times with lysis buffer and resuspended in 2× SDS loading buffer. Proteins were resolved by SDS-PAGE as previously described [45]. Autoradiographs were quantified using Andor IQ software (Andor, Belfast, UK).

### In vitro kinase assay

Kinases assays were performed as previously described [45]. Immune complexes were washed three times with lysis wash buffer, once with LiCl wash (0.5 M LiCl and 20 mM Tris pH 8.0) and once with kinase wash buffer (50 mM Tris–HCl pH 7.5, 10 mM MgCl_2_, and 1 mM DTT), then incubated in kinase reaction buffer (2.5 mM HEPES pH7.4, 50 mM MgAc, 0.5 mM ATP, 40 mM MOPs, pH 7.0 and 1 mM EDTA) containing 1 μCi/μl of [γ-^32^P] ATP together with Histone H1 or recombinant GST-β-catenin (Roche) for 30 min at 30 °C. The reaction was stopped by adding SDS-page loading buffer. Autoradiographs and Western blots were quantified using Andor IQ software (Andor, Belfast, UK) and the level of phosphorylation normalized to protein levels.

### GST pulldown

GST proteins were purified from BL21-A1 bacteria (Invitrogen) as previously described [2]. Cells were lysed in NP-40 lysis buffer (0.5 % NP-40, 50 mM Tris–HCl, pH 7.6, 30 mM sodium pyrophosphate, 150 mM NaCl, 0.1 mM EDTA, 50 mM NaF, 1 mM Na_3_VO_4_, 1 mM PMSF, 10 μg/ml leupeptin, 1 μg/ml aprotinin, and 1 mM DTT). Lysates were pre-cleared by incubation with GST-coupled Glutathione Sepharose 4 Fast Flow beads (Amersham) for 1 h at 4 °C, then incubated with the GST fusion protein beads for 2 h at 4 °C, collected by centrifugation, washed three times with lysis buffer, and resuspended in 2 × SDS loading buffer. Proteins were resolved by SDS-PAGE as previously described [45]. Autoradiographs were quantified using Andor IQ software (Andor, Belfast, UK).

## Electronic supplementary material

Below is the link to the electronic supplementary material.

Supplementary material 1 (PDF 6650 kb)
