# Supplementary material for: A PAK6–IQGAP1 complex promotes disassembly of cell–cell adhesions
Source: Cell Mol Life Sci. 2013 Dec 19;71(14):2759–73. doi: 10.1007/s00018-013-1528-5 (PMC4059965; doi:10.1007/s00018-013-1528-5)
Supplement: Supplementary file 1 — Supplementary material 1 (PDF 6650 kb) [file 18_2013_1528_MOESM1_ESM.pdf]

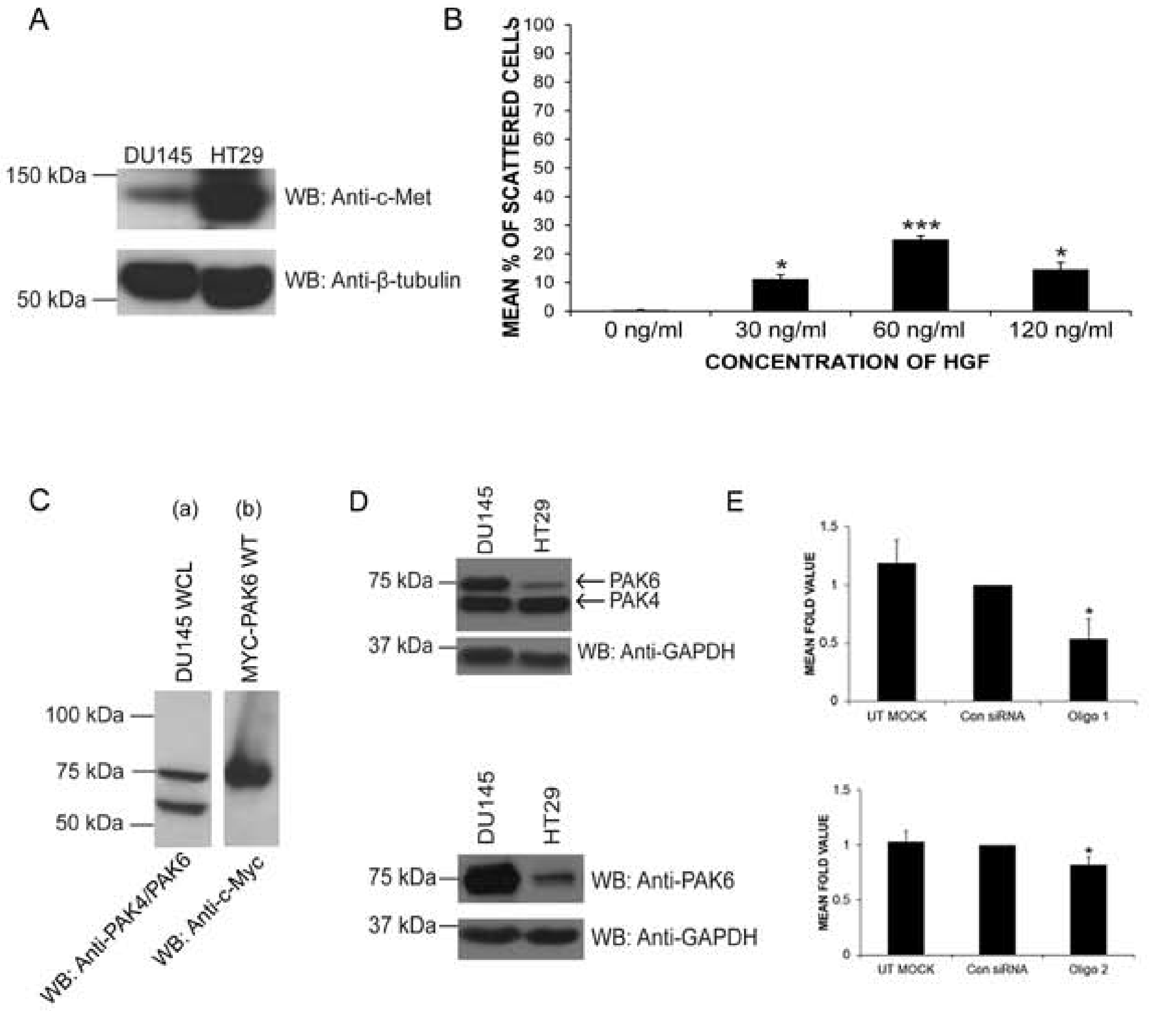

Figure S1 A) DU145 and HT29 whole cell lysates were immunoblotted for c-Met expression and for β-tubulin as a loading control. **B)** The HGF-induced scatter assay was conducted using HT29 cells. Cells were seeded onto glass coverslips and serum-starved for 24 hours prior to 24 hours HGF stimulation at varying concentrations. Cells were then fixed and stained for F-Actin. Cells were counted and cell counts of serum-starved versus serum-starved plus HGF were compared. Scattered = loss of cell: cell junctions and single cells with an elongated migratory phenotype. The mean % of scattered cells and the standard error of the mean were calculated over 3 independent experiments. Statistical significance compared with minus HGF cells was calculated using Student's *t*-test; *, $P < 0.05$ ***, $P < 0.0005$. **C)** DU145 whole cell lysate (WCL) was immunoblotted for PAK4/PAK6 expression using an anti-PAK4 antibody that recognises PAK6 (anti-PAK4/PAK6 antibody). (b) HEK293 cells expressing WT MYC-PAK6 were lysed and the lysate immunoblotted for PAK6 expression using an anti-c-Myc antibody. **D)** Whole cell lysates for the indicated cell lines were immunoblotted using the anti-PAK4/PAK6 antibody, an anti-PAK6 specific antibody as indicated. Lysates were also immunoblotted for GAPDH as a loading control. Data are representative of 3 independent experiments.

E) the mean fold value and the standard error of the mean were calculated over 3 independent experiments. Statistical significance compared between Con siRNA and UT Mock or PAK6 siRNA (Oligo 1 or 2) was calculated using Student's t-test: *, $P < 0.05$,

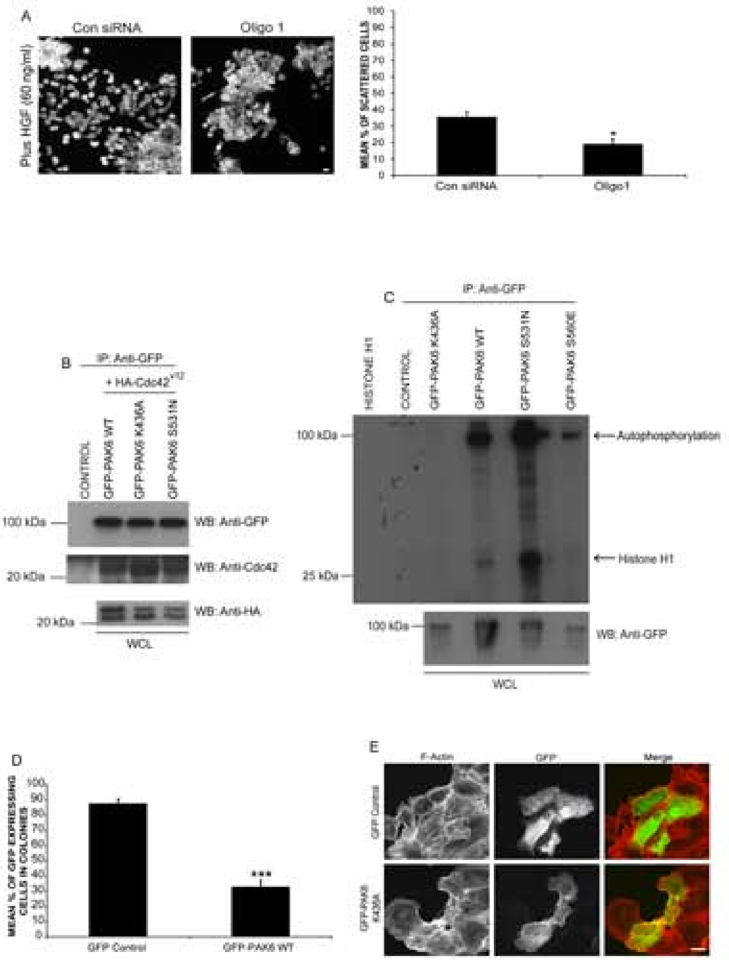

Figure S2 A) HT29 cells were transfected with con or PAK6 siRNA (oligo 1). Following 72 hours, cells were serum-starved, stimulated with HGF for 24 hours, fixed, stained for F-Actin and scored for cell scattering.. **B)** HEK293 cells were co-transfected with the indicated GFP-PAK6 mutants and HA-Cdc42^{V12}. PAK6 was immunoprecipitated using an anti-GFP antibody. IPs were immunoblotted using anti-GFP and anti-Cdc42 antibodies. WCL were immunoblotted using an anti-HA antibody C) GFP-PAK6^{wt} or derivatives was immunoprecipitated from cells and used in an in vitro kinase assay with histone H1 as a substrate. **D)** HT29 cells were transfected with GFP control or GFP-PAK6^{wt} serum-starved cells were fixed and stained for F-Actin. The % of GFP expressing cells present within a colony was calculated. **D)** DU145 cells were transfected with GFP control or GFP-PAK6^{K36A}. Ccells were serum-starved for 24 hours, fixed and stained for F-Actin. bar = 10 µm Statistical significance was calculated using Student's *t*-test; *, $P < 0.05$. The mean values and the standard error of the mean were calculated over 3 independent experiments. The blots shown are representative of 3 independent experiments.

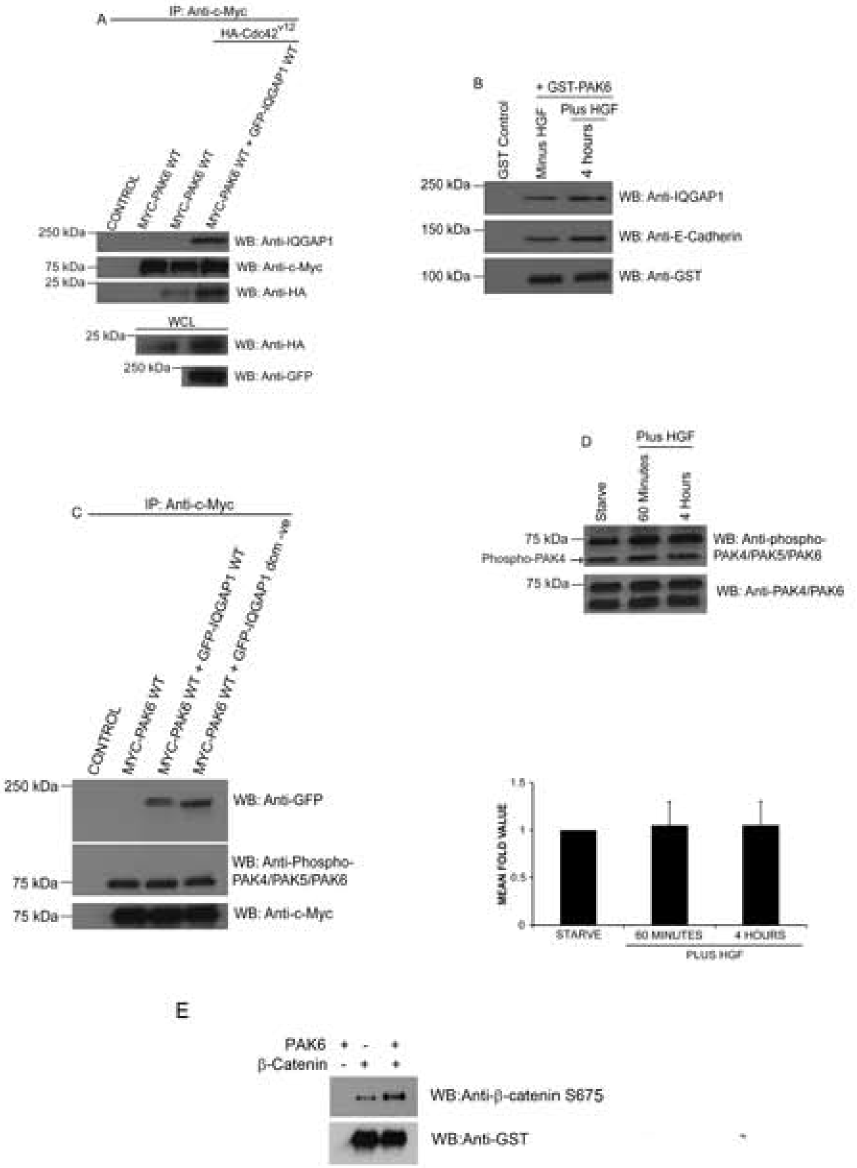

Figure S3 A) HEK293 cells were transfected with MYC-PAK6^{wt}, GFP-IQGAP1^{wt} and HA-Cdc42^{v12} as indicated. The cells were lysed and MYC-PAK6^{wt} was immunoprecipitated using an anti-c-Myc antibody. Samples were immunoblotted with an anti-GFP antibody, an anti-c-Myc antibody and an anti-HA antibody. Whole cell lysates (WCL) were immunoblotted using anti-HA and anti-GFP antibodies as loading controls. The blots shown are representative of 3 independent experiments. In (A) bar = 10 µm.**B)** HEK293 cells were transfected with WT MYC-PAK6, or co-transfected with WT MYC-PAK6 and WT GFP-IQGAP1 or dominant negative (dom –ve) GFP-IQGAP1. The cells were lysed and WT MYC-PAK6 was immunoprecipitated using an anti-c-Myc antibody (IP) from cell lysates. The samples were immunoblotted for GFP-IQGAP1 using an anti-GFP antibody and also for WT MYC-PAK6 using an anti-c-Myc antibody as a loading control. The IP samples were immunoblotted for levels of PAK6 autophosphorylation at serine 560 using a phospho-PAK4/PAK5/PAK6 antibody. C) GST-PAK6 or GST was used in a pulldown from serum starved or HGF stimulated DU145 cell lysates as indicated. Pull down samples were immunoblotted for endogenous IQGAP1 and endogenous E-cadherin. D) DU145 cell lysates were probed for the level of phospho-PAK6 S560 following prolonged HGF stimulation. Autoradiographs were analysed by densitometry and the phospho-signal was normalised to total protein. Representative of 3 independent experiments.
E) in vitro kinase assay using HIS-PAK6 and GST-β–catenin. Samples were probed for phospho-serine 675 and GST as a loading control

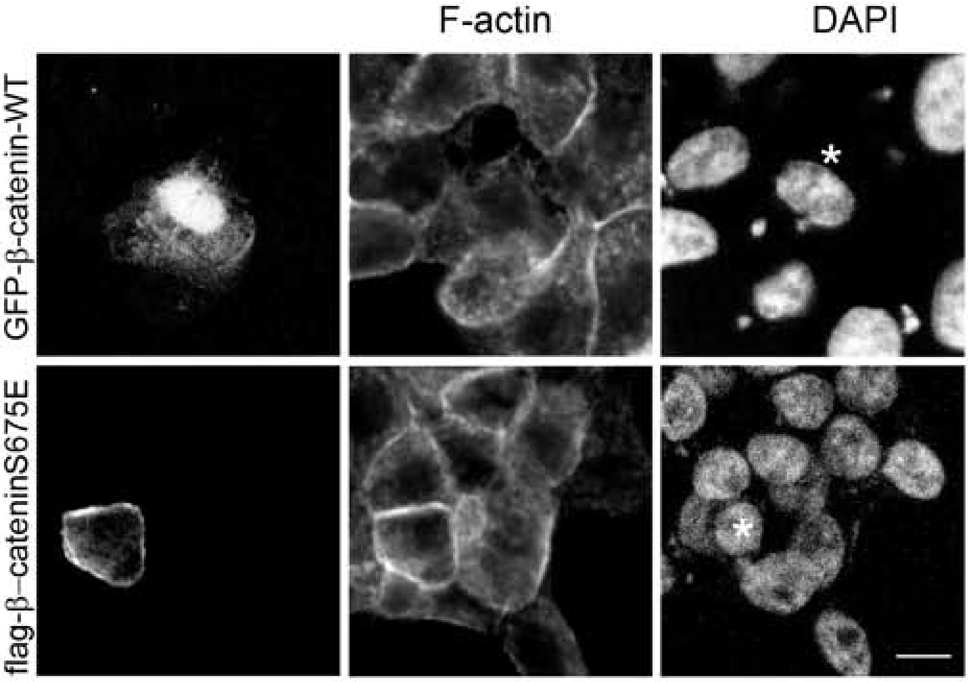

Figure S4. Capan-1 cells were transfected with GFP-β–catenin-WT or flag-β-cateninS675A. Cells were fixed and stained for DAPI, F-Actin and flag-tag where required.Representative of 3 independent experiments. bar = 10 µm.
